# Berberine Attenuates Glucocorticoid-Induced Bone Loss in Mice: Associated with the Gut Microbiota–Glycerophospholipid Metabolic Axis

**DOI:** 10.3390/foods15081325

**Published:** 2026-04-10

**Authors:** Suzhen Chao, Shengyuan Li, Jimin Zhong, Xinyi Peng, Yang Li, Min Shi, Xing Hu, Bo Liu

**Affiliations:** 1 School of Pharmacy, Jiangxi University of Chinese Medicine, Nanchang 330004, China; chaosuzhen@jxutcm.edu.cn (S.C.); lishengyuan2024@163.com (S.L.); zhongjimin@jxutcm.edu.cn (J.Z.); pengxinyi23@jxutcm.edu.cn (X.P.); ly2044@126.com (Y.L.); 2 School of Pharmacy, Nanchang Medical College, Nanchang 330052, China; 3 Key Research Laboratory of Prevention and Treatment of Geriatric Diseases with Chinese Medicine, Nanchang 330004, China; 4 School of Life Sciences, Jiangxi University of Chinese Medicine, Nanchang 330004, China; flea_sh@126.com; 5 State Key Laboratory of Food Science and Resources, Nanchang University, Nanchang 330047, China; 6 Jiangxi Provincial Key Laboratory of Agrofood Safety and Quality, Nanchang University, Nanchang 330047, China

**Keywords:** berberine, bone loss, gut microbiota, glycerophospholipid metabolism

## Abstract

Dietary supplementation with functional nutrients is a safe strategy to improve bone health. This study aimed to investigate the ameliorative effects of Berberine (BBR) on dexamethasone-induced bone loss in mice and its potential mechanisms. Micro-CT, histological staining, ELISA and Western blot were employed to evaluate BBR’s skeletal benefits; 16S rRNA sequencing, serum metabolomics and correlation analysis were used to explore its regulatory mechanisms. In vivo experiments showed that BBR improved bone mineral density and trabecular microarchitecture, and upregulated osteogenic markers (COL1 and BMP2). Intestinal bacterial sequencing showed that BBR altered gut bacterial composition, increasing the abundance of *Desulfovibrio* and *Bacteroides* while decreasing opportunistic pathogens. BBR also modulated bacterial richness, evenness, and community stability. Serum metabolomics identified 107 BBR-reversed differential metabolites; of these, 33.64% were lipids and lipid-like molecules, which were mainly involved in glycerophospholipid metabolism. Further correlation analysis revealed that BBR-enriched *Desulfovibrio* was linked to pathway R04864, producing a key glycerophospholipid metabolite positively correlated with bone mass parameters. Overall, these findings suggest that the attenuation of bone loss by BBR may be associated with alterations in the gut microbiota–glycerophospholipid metabolic axis, supporting its potential as a functional food ingredient for bone health.

## 1. Introduction

A robust skeletal system is essential not only for structural support and locomotion, but also plays a key role in important physiological functions such as mineral homeostasis and organ protection [1]. With the increasing prevalence of an aging population, bone loss has become a severe public health challenge, a prime example being osteoporosis (OP). This condition is primarily defined by systemic bone loss and the breakdown of trabecular structures, which ultimately elevates susceptibility to fractures [2]. Preserving skeletal integrity relies on the strict coupling of osteoclast-mediated catabolism and osteoblast-induced bone anabolism [3]. Long-term or high-dose use of glucocorticoids (GCs) is the main cause of secondary bone loss, which is clinically referred to as glucocorticoid-induced OP (GIOP) [4]. GCs such as dexamethasone (DEX) can severely disrupt this metabolic balance by inhibiting the function of osteoblasts and causing significant bone loss.

Currently, intervention strategies for bone health mainly encompass pharmacological treatments and basic nutritional supplementation. While conventional pharmacotherapies exhibit definite efficacy, their prolonged clinical use often leads to various adverse complications, ranging from gastrointestinal disturbances to osteosarcoma and potential cardiovascular disease risks [5,6]. Similarly, although basic nutritional supplementation (e.g., calcium agents and vitamin D) provide essential substrates for bone health, they are often limited by single-target mechanisms and thus can hardly fundamentally reverse bone loss caused by metabolic dysregulation [7]. In contrast, the dietary intake of functional nutritional ingredients shows unique potential. Natural functional factors often modulate the overall metabolic microenvironment through multi-target and multi-pathway synergistic regulatory mechanisms, thereby partially compensating for the limitations of single-nutrient supplementation in rectifying bone loss caused by complex metabolic disorders [8]. Consequently, exploring safe and efficacious natural dietary functional factors to maintain bone homeostasis via daily intervention constitutes a central focus in the fields of food science and nutrition [9].

Extracted from numerous edible and medicinal plants, berberine (BBR) is a well-known isoquinoline alkaloid distributed across multiple families, including Berberidaceae, Ranunculaceae, Menispermaceae and Rutaceae. Representative species include *Berberis vulgaris*, *Hydrastis canadensis*, *Zanthoxylum* spp., *Coptis chinensis* Franch. and *Phellodendron amurense* Schneid [10,11,12]. Within recommended dosage ranges, BBR has demonstrated a favorable safety profile and clinical tolerance [13]. It exhibits remarkable efficacy in regulating glucose and lipid metabolism, while also functioning as a robust modulator of oxidative stress and inflammatory cascades [14,15]. As a highly promising functional nutritional ingredient, BBR operates differently from the “confrontational” mechanisms of conventional pharmacotherapies. Instead, it functions primarily through “systemic regulation”. In particular, BBR has unique metabolic kinetics characteristics of low oral absorption rate and long intestinal residence time. This feature enables it to have a persistent interaction with the gut microbiota, providing a solid physiological basis for investigating the gut–bone axis [16].

Recently, the conceptual framework of the gut–bone axis has emphasized how gut microbiota actively govern the dynamics of bone turnover [17]. An expanding body of literature highlights that enteric dysbiosis is inextricably linked to disruptions in lipid metabolism, especially glycerophospholipid metabolism, which is pivotal to the signaling cascades that control the activity of osteoblasts and osteoclasts [18]. Consequently, targeting the remodeling of the “gut microbiota–glycerophospholipid metabolism” network is regarded as a promising strategy for attenuating bone loss. However, despite established evidence of BBR’s osteoprotective properties [19] and its modulatory influence on the gut microbiota [20], the specific mechanisms, particularly whether BBR exerts its bone-protective effects by reshaping the gut microbiota to specifically restore the host glycerophospholipid metabolic profile, remain to be fully elucidated.

To accurately simulate the complex interplay between clinical bone loss and systemic metabolic shifts, a DEX-induced mouse model was employed, as it effectively recapitulates the bone loss phenotype characterized by compromised bone remodeling and disrupted physiological homeostasis. Building upon this established model, this study aimed to comprehensively characterize BBR’s regulatory impact on intestinal microbial architecture and systemic lipidomic signatures. We utilized a multi-omics strategy that merged 16S rRNA gene sequencing and untargeted metabolomics to explore the underlying mechanisms. The study may provide a robust scientific rationale for the development of novel functional food ingredients targeting skeletal homeostasis.

## 2. Materials and Methods

### 2.1. Reagents and Materials

BBR (CAS: 2086-83-1, Cat. No. S27357, purity: 95%) was obtained from Yuanye Bio-Technology Co., Ltd. (Shanghai, China). DEX injection (batch: 20240601) was supplied by Ruicheng Kelong Veterinary Medicine (Yuncheng, China), while alendronate sodium tablets (batch: Y011031) were from MSD Pharma (Hangzhou, China). The analytical kits, including BCA Protein Assay Kit (QI220551) and ELISA sets for CTX1 (C-terminal telopeptide of type I collagen) (MM-47264M1) and P1NP (N-terminal propeptide of type I procollagen) (MM-45130M1), were from Thermo Fisher Scientific (Shanghai, China) and Jiancheng Bioengineering Institute (Nanjing, China), respectively. Specific antibodies against COL1 (ab270993), BMP2 (A27101) and β-actin (GB15003-100) were supplied by Abcam (Cambridge, UK), ABclonal Technology (Wuhan, China) and Servicebio (Wuhan, China). Additionally, Loading buffer (DL101) was from TransGen Biotech Co., Ltd. (Beijing, China); Dual-color pre-stained protein Marker (WJ102) and Protein-free Rapid Blocking Buffer (PS108P) were provided by Shanghai Yamei Biomedical Technology Co., Ltd. (Shanghai, China); SDS-PAGE Rapid Electrophoresis Buffer (P0562-10L) and Western Transfer Buffer (P0021B) were purchased from Beyotime Biotechnology Co., Ltd. (Shanghai, China); Primary Antibody Diluent (P0256) was obtained from Beyotime Biotechnology Co., Ltd. (Shanghai, China); TBST Buffer (T1081) was supplied by Solarbio Science & Technology Co., Ltd. (Beijing, China); and ECL Ultra-sensitive Luminescent Solution (BMU102-CN) was purchased from Abbkine Scientific Co., Ltd. (Wuhan, China).

### 2.2. Network Pharmacology Analysis

BBR’s molecular structure was retrieved from PubChem, and potential targets were predicted via SwissTargetPrediction, SEA, and STITCH, with duplicates removed to establish a candidate profile. Simultaneously OP-related targets were consolidated from OMIM, GeneCards and DisGeNET. The intersection between BBR and OP targets was identified using Venny 2.1 to define the core therapeutic gene set. Subsequently, the PPI network was visualized using Cytoscape 3.9.1, from which the top 10 hub targets were isolated based on Degree centrality. Functional insights were gained through GO and KEGG enrichment analyses via the DAVID platform, with a significance threshold set at *p* < 0.05 and FDR correction applied, and visualization performed on the Bioinformatics online tool. Binding affinities between BBR and hub targets were ultimately validated by the CB-DOCK2 online molecular docking tool.

### 2.3. Animal Experiments

#### 2.3.1. Model Establishment and Grouping

A total of 24 healthy, SPF male C57BL/6J mice, at an age of 3 months and averaging 25 ± 2 g in body weight, were obtained from Huachuang Xinnuo (Jiangsu) Pharmaceutical Tech. Co., Ltd., China. (Registration code SCXK (Su) 2020-0009). All mice were raised in a temperature-controlled (25 ± 2 °C) and humidity-controlled (50 ± 10%) facility with a 12 h light/dark cycle. Animals were co-housed in standard cages (6 mice per cage, grouped by treatment). All mice had ad libitum access to a standard laboratory maintenance diet and sterile water. Autoclaved wood shavings were used as bedding, and no additional environmental enrichment was provided to maintain experimental consistency. All procedures were performed in accordance with the institutional guidelines for animal care. The animal study protocol was approved by the Laboratory Animal Ethics Committee of Jiangxi University of Chinese Medicine (Approval No. JZLLSC-20240566).

After a one-week acclimatization period, mice were individually assigned random numbers and divided into four experimental groups (n = 6 per group) by simple randomization (drawing lots): normal control group (Control), model group (Model), BBR intervention group (BBR), and positive drug control group (Positive control). The administration vehicle used was 0.9% NaCl (physiological saline) for all treatments. To establish a GCs-induced bone loss model, mice in the Model, BBR, and Positive control groups received daily intraperitoneal (i.p.) injections of DEX sodium phosphate at a dose of 50 mg/kg body weight [21], with a final concentration of 5 mg/mL and an administered volume of 10 mL/kg. Control mice received an equal volume of saline via i.p. injection. Concurrently, mice in the Control and Model groups were given an equivalent volume of physiological saline via intragastric gavage (i.g.) (10 mL/kg), without additional functional ingredients. Mice in the BBR group received BBR (suspended in saline at 10 mg/mL) by gavage at 100 mg/kg (10 mL/kg) [22]. The Positive control group received alendronate sodium (0.2 mg/mL in saline) by gavage at 2 mg/kg (10 mL/kg) [23]. All treatments were performed daily for 6 weeks using 1 mL sterile syringes for i.p. injections and standard gavage needles for oral administration.

The body weight of each mouse was measured once a week to monitor their general physiological status. The inclusion criteria for this study required mice to be in good health with normal activity at the beginning of the experiment. Exclusion criteria were pre-defined as follows: (1) accidental death during the intervention period; (2) severe weight loss; (3) visible signs of distress, infection, or injury that could interfere with the experimental outcomes. Throughout the 6-week experimental period, no mice met the exclusion criteria, and all animals were included in the final statistical analysis. At the end of the study, the mice were anesthetized with isoflurane and euthanized via cervical dislocation for sample collection. To ensure objective evaluation, researchers involved in the subsequent assessment of related indicators remained blinded to the group allocations, identifying samples solely by their numerical codes.

#### 2.3.2. Serum Collection and Preservation

Mouse blood samples were allowed to clot naturally at room temperature for 1 h, followed by centrifugation at 3000 r/min for 10 min at 4 °C using a TGL-16.5M high-speed desktop refrigerated centrifuge (Luxiangyi Instrument Co., Ltd., Shanghai, China). We carefully aspirated the upper layer of serum, aliquoted it into pre-cooled EP tubes according to the experimental requirements, and immediately stored it in a −80 °C ultra-low temperature refrigerator for use.

#### 2.3.3. Micro-CT Analysis

Micro-CT analysis was performed in 3 randomly selected mice per group. Femoral samples were fixed and subsequently scanned using a high-resolution micro-CT system (SkyScan 1276; Bruker, Rheinstetten, Germany). During the scanning process, operational configurations were optimized with specific source settings (60 kV, 200 μA), capturing data at an isotropic voxel size of 6.55 μm over a half-circle trajectory. Subsequent to image acquisition, volumetric 3D reconstruction was performed via NRecon software (V1.7.4.2, Bruker, Germany). The region of interest (ROI) for trabecular bone was strictly defined at the distal femoral metaphysis, commencing 0.5 mm proximal to the distal growth plate and extending proximally for 150 consecutive slices (approximately 1 mm in length). Finally, CT Analyzer software (Version 1.20.3.0) was utilized to quantify key microarchitectural indices within the defined ROI, specifically focusing on bone mineral density (BMD), bone volume fraction (BV/TV) and trabecular morphology (Tb.N and Tb.Sp).

#### 2.3.4. H&E Staining

Femoral samples were initially immersed in 4% paraformaldehyde for 48 h at room temperature for stabilization, followed by a decalcification process in 10% EDTA buffer (pH 7.4) at ambient temperature. After sufficient decalcification, the samples were dehydrated through a graded series of ethanol solutions (50%, 70%, 85%, 95%, and 100%) and cleared in xylene before being embedded in paraffin blocks. Five-micrometer-thick cross-sections were obtained and transferred onto adhesive slides. The prepared slides underwent routine H&E staining: sections were deparaffinized, stained with hematoxylin for 5 min, differentiated with 1% hydrochloric acid alcohol, blued with 0.6% ammonia water, and counterstained with eosin for 5 min. Finally, the slides were dehydrated, cleared, and mounted with neutral resin to evaluate femoral histopathology via bright-field microscopy using a MD1000 microscope (Leica Microsystems Ltd., Wetzlar, Germany).

#### 2.3.5. Serum ELISA

The levels of biochemical markers (CTX-1 and PINP) were quantified by adding mouse serum supernatant to the ELISA microplate wells. Following a 60 min incubation period at 37 °C, the plate underwent 5 wash cycles to eliminate any non-specifically bound components. The detection phase involved the addition of a horseradish peroxidase-linked secondary antibody, which was then incubated for 30 min. The plate was washed again to remove unbound secondary antibody. The chromogenic reaction was initiated by adding the substrate solution and allowing it to develop in the dark for 15 min. The enzymatic process was halted using a stop solution, after which the OD was measured at 450 nm using a microplate reader (FlexA-200HT, Aosheng Instrument Co., Ltd., Hangzhou, China). Finally, the systemic levels of the two bone turnover markers were determined by referencing the established standard curves.

#### 2.3.6. Western Blot Analysis

Total protein was extracted from the femoral samples following a mechanical pulverization process. Specifically, the femurs were homogenized in 150 µL of RIPA lysis buffer supplemented with protease inhibitors using a Scientz-24 High-Throughput Tissue Grinder (Ningbo Xinzhi Biotechnology Co., Ltd., Ningbo, China), then placed in a 4 °C refrigerator for lysis for 30 min, followed by centrifugation at 12,000 r/min for 10 min to separate the supernatant protein. Protein concentration was assessed via a standard BCA assay. The protein samples were mixed with 6× Loading Buffer at a ratio of 5:1, then denatured by boiling at 95 °C for 10 min. Equivalent protein aliquots (20 μg per well) underwent electrophoretic separation on 10% SDS-PAGE gels at a constant voltage of 100 V for 90 min, and were subsequently transferred to PVDF membranes. The transfer conditions were set as follows: constant current of 250 mA, and the transfer time was optimized according to the molecular weight of the target proteins: 90 min for COL1 and 60 min for BMP2. After a 30 min rapid blocking phase, the blots were probed with primary antibodies (1:1000, 4 °C) overnight. Following serial washes in 1× TBST (3× 10 min), the membranes were exposed to corresponding secondary antibodies at ambient temperature for 1.5 h. Immunoreactive bands were developed using an ECL reagent and recorded by a chemiluminescence unit, with band intensities quantified via ImageJ software (Version 1.46r) and standardized against the β-actin loading control.

### 2.4. Gut Bacterial Analysis

To profile the intestinal bacterial communities, 16S rRNA gene sequencing was executed on cecal contents (n = 6 per group; including Control, Model, and BBR groups) by Applied Protein Technology Co., Ltd. (APTBIO, Shanghai, China). Briefly, the cecal contents were collected immediately after sacrifice under sterile conditions, snap-frozen in liquid nitrogen, and stored at −80 °C. Then, the CTAB protocol was utilized to isolate total genomic DNA, followed by specific PCR amplification targeting the V3-V4 segments of the 16S ribosomal RNA. Following library preparation, paired-end reads were acquired utilizing the Illumina NovaSeq 6000 system. The resulting raw datasets underwent bioinformatics processing via the QIIME2 pipeline, encompassing quality filtering, sequence denoising, pair assembly, and chimera depletion, ultimately yielding Amplicon Sequence Variants (ASVs). Taxonomic annotation was performed against the SILVA 16S rRNA database (version 138) using the classify-sklearn algorithm with default parameters. Based on the ASVs, downstream analyses were conducted, including α-diversity (Shannon, Simpson and Chao1 indices), β-diversity (PCA, PCoA, NMDS and ANOSIM), bacterial community structure, species difference analysis and functional prediction (KEGG and COG).

### 2.5. Untargeted Serum Metabolomics Analysis

Serum metabolic profiling was conducted by APTBIO (Shanghai, China) (n = 6 per group; Control, Model, and BBR groups). Briefly, 100 μL of serum was thawed at 4 °C and extracted using a precooled mixture of methanol:acetonitrile:water at a volume ratio of 2:2:1 (*v*/*v*/*v*), followed by low-temperature sonication for 30 min and centrifugation at 14,000× *g* for 20 min at 4 °C. The resulting supernatants were collected, concentrated via vacuum drying, and subsequently reconstituted in an acetonitrile/water solution for analysis. Chromatographic resolution was attained using the 1290 UHPLC model (Agilent Technologies, Santa Clara, CA, USA), which was integrated with an ACQUITY UPLC BEH Amide separation column (Waters). Gradient elution was performed using water containing ammonium acetate/ammonia and acetonitrile as the mobile phases. An AB Triple TOF 6600 platform, configured with an electrospray ionization (ESI) source, was utilized for mass spectrometry (MS) data collection via a data-dependent acquisition (DDA) strategy. To ensure system stability and data reliability, the sample injection order was randomized, and QC samples were interspersed throughout the run. XCMS software (Version 3.12.0) was employed for data processing of the raw MS files. Subsequently, multivariate statistical analyses (PCA and OPLS-DA) and metabolite pathway enrichment analysis based on the KEGG database were performed.

### 2.6. Correlation Analysis

To determine the correlations between intestinal bacteria, serum metabolites, and skeletal metabolism-related indicators, R software (v4.5.0) was utilized to conduct comprehensive statistical integration. Spearman’s rank correlation coefficients were calculated using the cor.test function (R base stats package) with exact = FALSE for approximation. To minimize the risk of false-positive results in multi-dimensional datasets, *p* values were adjusted for multiple testing using the Benjamini–Hochberg false discovery rate (FDR) method. Quantitative data for core skeletal parameters (BMD, BV/TV, Tb.N, Tb.Sp), dominant gut genera (top 20 with relative abundance), and the 107 significantly differential metabolites were standardized using Z-score transformation (z = (x − μ)/σ) to eliminate technical biases arising from different measurement scales. The standardized values were then merged to construct a unified multi-dimensional dataset. Correlations with an FDR-adjusted *p* value (Q-value) < 0.05 were considered statistically significant (* *p* < 0.05, ** *p* < 0.01). For subsequent visualization, the top 10 genera and metabolites showing the strongest associations with bone parameters (ranked by smallest FDR-adjusted *p* values) were selected, together with the four bone parameters, to construct the correlation heatmap and the correlation data were exported for network visualization in Cytoscape (v3.9.1). Correlation matrices were visualized using the pheatmap package in R.

### 2.7. Metabolite Tracing and Sankey Network Analysis

The top 25 differential metabolites most strongly correlated with bone metabolism parameters (identified above) were selected for source tracking analysis using the MetOrigin platform to clarify their origins. Concurrently, by integrating the 16S rRNA sequencing data and untargeted metabolomics data, a Sankey network diagram was generated via MetOrigin. This analysis further focused on the key reaction R04864 (PAPE synthesis) within the glycerophospholipid metabolism pathway to determine its association with core microbiota and clarify the functional contribution of core microbiota to PAPE synthesis.

### 2.8. Statistical Analysis

Data processing and computational evaluations were executed utilizing the SPSS 27.0 environment. Experimental results are documented as the mean alongside the standard deviation (mean ± SD). Before performing one-way ANOVA, the normality of data distribution was verified using the Shapiro–Wilk test, and the homogeneity of variances was confirmed by Levene’s test. All data met the requirements for parametric analysis. To determine the significance of variations across multiple cohorts, single-factor ANOVA was implemented. If the ANOVA showed significant differences, further pairwise comparisons were conducted using the LSD test. Inter-variable associations were quantified via Spearman’s rank analysis. All graphical representations were rendered utilizing GraphPad Prism (version 8.0.2), with alpha levels strictly set at *p* < 0.05 and *p* < 0.01 to denote statistical and high significance, respectively.

## 3. Results

### 3.1. Network Pharmacology Predicts Targets and Pathways by Which BBR Ameliorates Bone Loss

**Figure 1:**
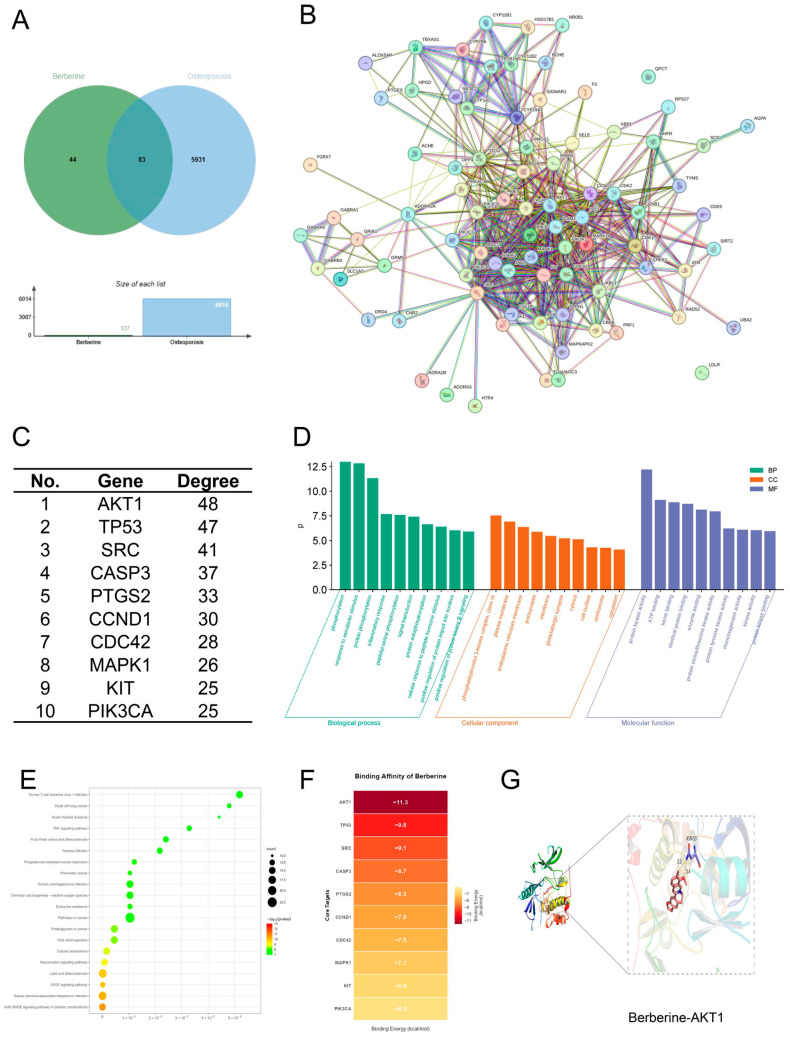
Network pharmacology and molecular docking analysis of Berberine (BBR) targeting osteoporosis (OP). (**A**) Venn diagram. (**B**) Protein-protein interaction (PPI) mapping of the shared genes. (**C**) Identification of the top 10 key targets. (**D**) Gene Ontology (GO) functional annotation. (**E**) Kyoto Encyclopedia of Genes and Genomes (KEGG) pathway mapping. (**F**) Heatmap of binding energies from molecular docking between BBR and core targets. (**G**) Molecular docking visualization of BBR with AKT1.

In this study, network pharmacology was initially employed to preliminarily explore the potential targets and related signaling pathways underlying the protective effects of BBR against bone loss. By integrating data from BBR target prediction databases and OP-related disease databases, we identified a total of 83 intersecting targets (Figure 1A). Topological analysis of the PPI network showed that AKT1, TP53 and SRC were the core targets exhibiting the highest degree values (Figure 1B,C).

As illustrated by the GO functional annotation (Figure 1D), the shared targets were enriched in biological modules associated with stress-induced cellular reactions and the regulation of protein phosphorylation. Regarding cellular components (CCs), the targets were highly concentrated in the phosphatidylinositol 3-kinase (PI3K) complex (comprising PIK3CA, PIK3CD, PIK3CB, PIK3R1, and PIK3CG), plasma membrane, and endoplasmic reticulum membrane. Given that phosphatidylinositol is a crucial glycerophospholipid serving as a core structural lipid in cell membranes and the endomembrane system, these findings preliminarily suggest that the ability of BBR to attenuate bone loss may be closely associated with glycerophospholipid metabolism and membrane lipid homeostasis. KEGG pathway enrichment analysis (Figure 1E) revealed that these targets were primarily involved in pathways such as “Lipid and atherosclerosis”, “AGE-RAGE signaling pathway”, and “TNF signaling pathway”.

To further verify the binding affinity between BBR and core targets, key proteins were selected for molecular docking simulations As shown in Figure 1F, all core targets displayed favorable binding affinity with BBR, yielding calculated binding free energies below −5 kcal/mol. Notably, BBR exhibited a relatively high docking score for AKT1 (binding energy: −11.3 kcal/mol) (Figure 1G).

### 3.2. BBR Attenuates Bone Loss In Vivo

**Figure 2:**
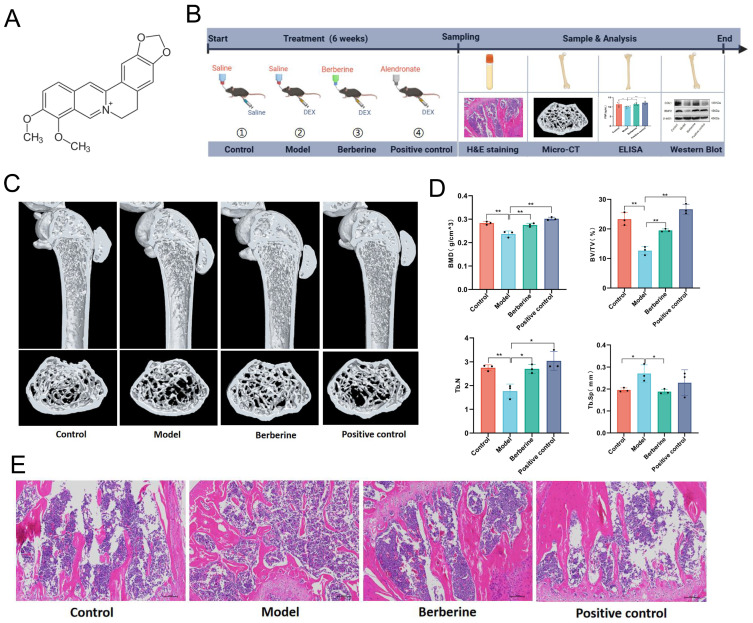
BBR ameliorates Dexamethasone (DEX)-induced bone loss in mice. (**A**) Structural formula of BBR. (**B**) Graphical summary of the in vivo animal protocol. (**C**,**D**) Micro-CT analysis of the femur (n = 3). (**E**) H&E staining of bone tissue. * *p* < 0.05 and ** *p* < 0.01.

To gain insights into the protective effects of BBR against DEX-induced bone loss, an in vivo mouse model was established. Subsequent analyses evaluated BBR-mediated improvements in bone mass, trabecular structure, and osteogenic activity (Figure 2A,B).

As illustrated in the micro-CT images (Figure 2C,D), DEX-exposed animals displayed substantial bone loss, characterized by decreased BMD, BV/TV and Tb.N, together with an enlarged Tb.Sp, relative to untreated healthy animals (*p* < 0.05). These findings confirmed that DEX treatment caused marked bone loss and structural impairment. However, BBR intervention effectively reversed these alterations and restored the related parameters toward normal status. These data demonstrated the bone-preserving effect of BBR. HE staining observations (Figure 2E) revealed that the Model group showed apparent structural alterations in osseous tissue, characterized by fewer and thinner bony trabeculae, along with expanded medullary areas compared to the Control group. Conversely, animals receiving BBR intervention exhibited trends toward improved trabecular microarchitecture, with relatively more continuous trabecular networks and decreased marrow spaces. These qualitative morphological features are consistent with the results obtained from micro-CT analysis, collectively reflecting the role of BBR in maintaining the integrity of the bone structure.

**Figure 3:**
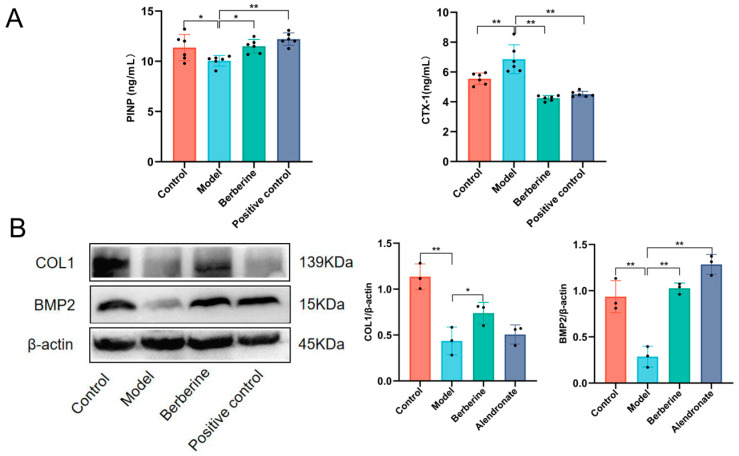
BBR upregulates osteogenic markers and promotes bone formation in DEX-induced bone loss mice. (**A**) Serum levels of C-terminal telopeptide of type I collagen (CTX-1) and procollagen type I N-terminal propeptide (PINP) were measured by ELISA (n = 6). (**B**) Protein expression of collagen type I (COL1) and bone morphogenetic protein 2 (BMP2) in bone tissue was detected by Western blot (n = 3). * *p* < 0.05 and ** *p* < 0.01.

ELISA results (Figure 3A) showed that P1NP concentrations were notably lower in DEX-treated animals than in untreated healthy counterparts (*p* < 0.05). However, BBR intervention notably raised P1NP levels (*p* < 0.05). Conversely, circulating CTX-1 concentrations were notably higher in DEX-exposed animals relative to their healthy counterparts (*p* < 0.05), whereas BBR intervention prominently lowered these values (*p* < 0.05). Collectively, these data suggest that BBR may alleviate bone loss by modulating the balance between bone formation and resorption, as indicated by the increased P1NP and decreased CTX-1 levels. Subsequently, Western blotting of bone formation markers (Figure 3B and Figure S1) showed that BBR treatment significantly restored the protein expression of COL1 and BMP2, which were suppressed by DEX induction (*p* < 0.05). These results indicate that BBR possesses the potential to promote bone formation by upregulating pivotal osteogenic factors, thereby contributing to the amelioration of bone loss.

### 3.3. BBR Regulates Gut Bacterial Composition

**Figure 4:**
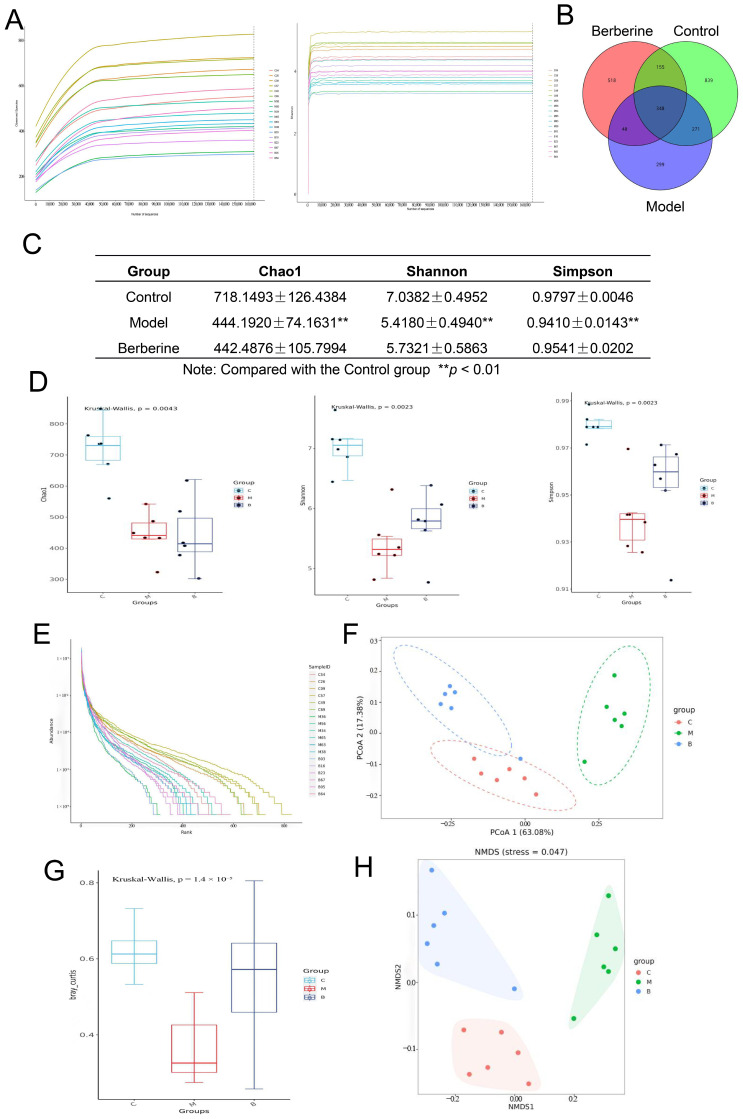
BBR alters gut bacterial composition in DEX-induced bone loss mice. (**A**) Rarefaction curves (**left**) and Shannon curves (**right**). (**B**) Venn diagram of amplicon sequence variants (ASV) clustering analysis. (**C**) α diversity indices (n = 6). (**D**) Box plots showing inter-group differences in α diversity indices: Chao1 (**left**), Shannon (**middle**), and Simpson (**right**). (**E**) Rank-abundance curves. (**F**) Principal coordinate analysis (PCoA). (**G**) Box plots of β diversity differences based on Bray–Curtis distance. (**H**) Non-metric multidimensional scaling (NMDS) analysis. In all panels, C, control group; M, DEX-treated model group; B, BBR-treated group. (applied to all figures).

To explore the modulatory role of BBR on the gut bacteria, cecal contents collected from all mice were subjected to 16S rRNA gene amplicon sequencing. Rarefaction curves confirmed that the sequencing depth was adequate to represent most bacteria species (Figure 4A). Analysis of shared and unique ASVs showed that DEX induction significantly reduced the number of ASVs in the gut bacteria (decreasing from 1613 in naive healthy animals to 958 in DEX-treated animals) (Figure 4B), suggesting a severe loss of species richness in the model mice. However, following BBR intervention, both the overall count of ASVs and the quantity of unique ASVs rebounded, indicating that BBR contributed to the restoration of the perturbed bacteria community structure.

The α diversity analysis further corroborated these findings. As depicted in Figure 4C,D, the induced pathology caused a significant decline in the Chao1, Shannon, and Simpson diversity scores (*p* < 0.01) when contrasted with normal controls. Such downregulations underscore a compromised state of bacteria proliferation and ecological stability in the gut environment. While the discrepancy between the BBR-treated and DEX-exposed groups failed to achieve statistical significance, all indices displayed an obvious upward recovery trend (Figure 4C–E). Regarding β diversity, Principal Coordinate Analysis (PCoA) found distinct separation in bacteria composition across all experimental groups (Figure 4F). Clear segregation in spatial distribution was observed between the healthy and model groups (PCoA1 = 63.08%), confirming that the modeling process caused a substantial shift in the overall bacteria structure. Notably, in the PCoA score plot, the BBR-treated animals formed a discrete cluster distinct from DEX-treated animals, and exhibited a tendency to converge with the healthy controls. This result was further supported by ANOSIM and NMDS analysis. ANOSIM was performed using the Bray–Curtis dissimilarity metric (*p* < 0.001), and the NMDS plot yielded a favorable stress value (Stress < 0.05; Figure 4G,H).

### 3.4. BBR Modulates Gut Bacterial Function

**Figure 5:**
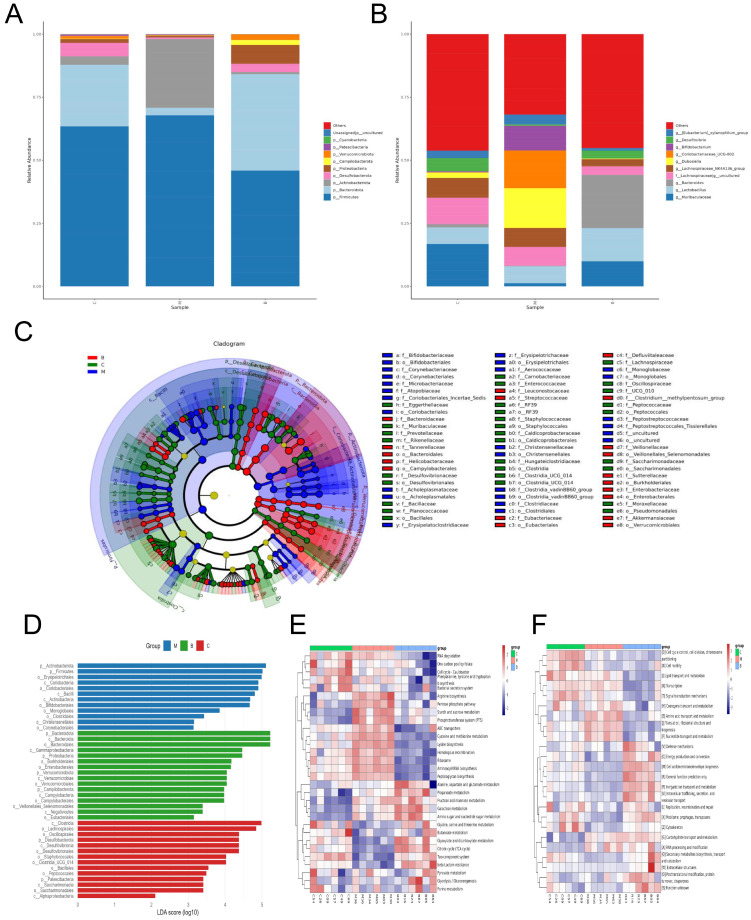
BBR reshapes gut bacterial structure and functional profiles. (**A**,**B**) Taxonomic composition of gut bacteria at the phylum (**A**) and genus (**B**) levels. (**C**) Phylogenetic mapping of differentially enriched taxa utilizing a LEfSe-based cladogram. (**D**) Histogram of LDA scores (LDA threshold > 3.0, *p* < 0.05). (**E**) KEGG functional prediction analysis. (**F**) Clusters of Orthologous Groups (COGs) functional classification.

Taxonomic analysis revealed that BBR significantly ameliorated gut bacterial dysbiosis induced by DEX. Taxonomic profiling at the phylum level (Figure 5A) found a severe depletion of Bacteroidota, Desulfobacterota and Pseudomonadata (formerly Proteobacteria) in the model animals, accompanied by an abnormal elevation in Actinobacteriota. BBR intervention effectively reversed these proportional imbalances. When assessed at the genus taxon (Figure 5B), BBR markedly normalized the relative proportion of bacterial taxa whose levels were diminished by glucocorticoid exposure, including *Desulfovibrio*, *Lactobacillus* and *Bacteroides*. Concurrently, it inhibited the overproliferation of opportunistic pathogens such as *Coriobacteriaceae_UCG-002* and *Dubosiella*.

Subsequent LEfSe profiling pinpointed distinct taxonomic biomarkers that discriminated between the cohorts (Figure 5C,D). Conversely, while the modeled animals exhibited an expansion of Actinobacteriota, BBR intervention markedly upregulated the abundance of beneficial commensals, notably Bacteroidota. This suggests that BBR may regulate the gut bacterial community by increasing the abundance of key beneficial bacteria. Furthermore, PICRUSt-based functional prediction found an adaptive remodeling of the bacterial metabolic potential (Figure 5E,F). KEGG prediction demonstrated that bacterial functions in DEX-treated animals were characterized by enhanced carbohydrate metabolism and membrane transport activity. However, BBR treatment significantly reversed this metabolic profile, shifting it toward energy metabolism (TCA cycle) and the biosynthesis of secondary metabolites.

### 3.5. BBR Modulates Serum Metabolic Profiles

**Figure 6:**
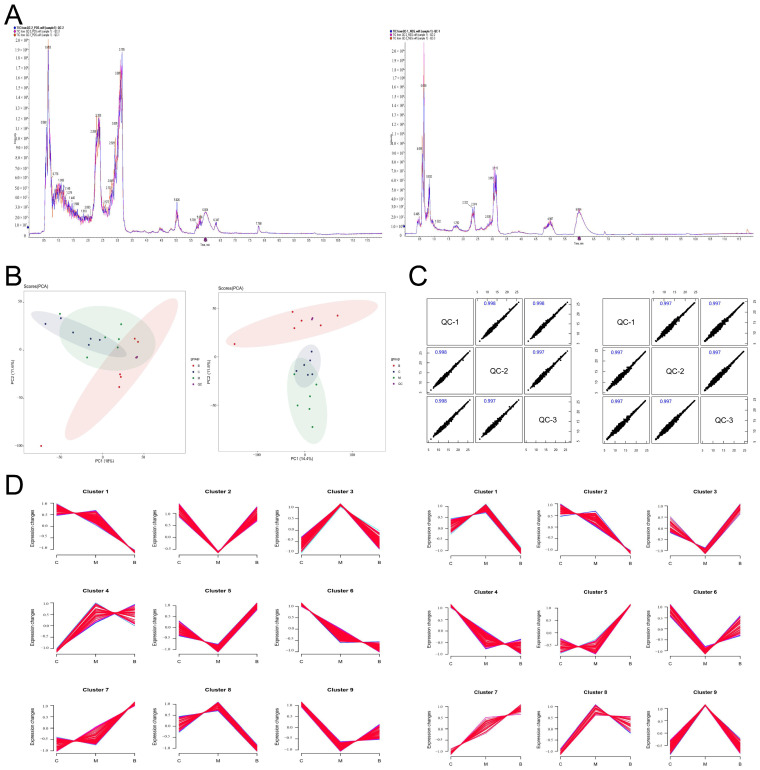
Untargeted metabolomics analysis of mouse serum. (**A**) Representative Total Ion Chromatograms (TICs) of the pooled QC. (**B**) Unsupervised principal component analysis (PCA) score distributions. (**C**) Inter-sample Pearson correlation matrices. (**D**) Trends of identified metabolites across different groups. Note: For all panels, data acquired under positive and negative ionization modes are displayed on the left and right, respectively.

To characterize the modulatory effects of BBR on the serum metabolic profile, we performed untargeted metabolomics analysis using UHPLC-Q-TOF MS. Assessment of Quality Control (QC) samples confirmed the high stability and data reliability of the instrument system (Figure 6A–C). PCA showed that glucocorticoid induction caused a significant shift in serum metabolic profiles, resulting in distinct spatial separation between the control and model groups. Furthermore, Fuzzy c-means clustering analysis found dynamic changes in metabolite patterns. Metabolites in Cluster 3 displayed a typical pattern: maintained at high levels in the healthy animals, markedly reduced in the DEX-treated animals, and effectively restored upon BBR administration (Figure 6D). These findings supported that BBR markedly counteracted the metabolic disturbances triggered by glucocorticoid exposure.

**Figure 7:**
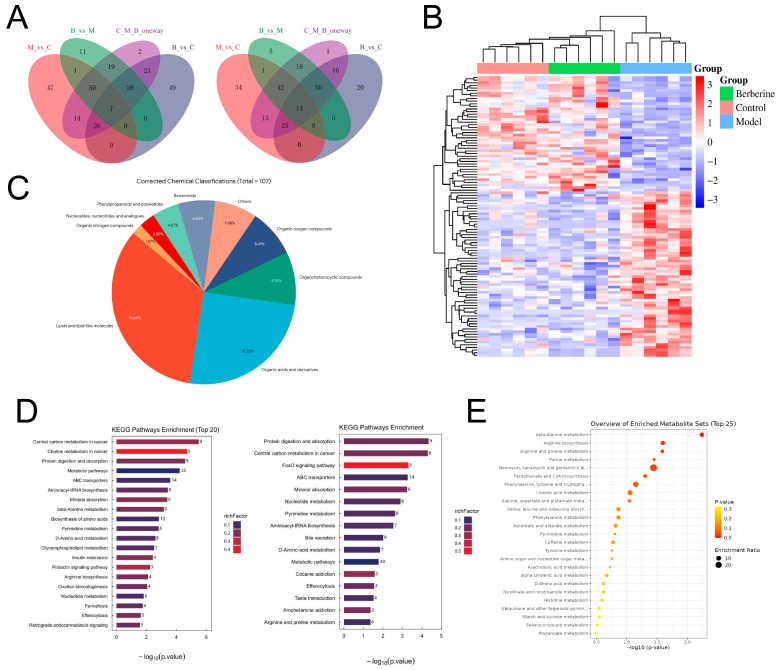
Analysis of serum differential metabolites and functions. (**A**) Venn diagram of overlapping significant differential metabolites. (**B**) Heatmap of 107 common differential metabolites. (**C**) Pie chart showing the classification distribution of characteristic metabolic markers. (**D**) KEGG pathway enrichment analysis. Model vs. Control (**left**); BBR vs. Model (**right**). (**E**) KEGG functional annotation mapping of the 107 characteristic metabolic markers.

For the identification of candidate metabolic biomarkers, OPLS-DA (Orthogonal Partial Least Squares Discriminant Analysis) was applied to distinguish metabolites with significant differences across cohorts (VIP > 1, *p* < 0.05). Relative to the healthy animals, 274 differential serum metabolites were perturbed in the DEX-treated animals. Following BBR intervention, 259 metabolites were regulated. Venn diagram analysis identified 107 specific metabolic markers that were significantly reversed by BBR (Figure 7A,B). Based on structural categorization (Figure 7C), lipid and lipidoid species constituted the largest proportion of these differential metabolites, representing 33.64%, followed by the class of organic acids alongside their derivatives. These observations suggest that the remodeling of the lipid metabolic profile is the most significant feature of BBR-mediated improvement of the serum metabolic microenvironment in the bone loss mouse model.

Functional annotation via the KEGG database showed that the metabolic variations distinguishing the diseased cohort from the healthy controls were predominantly concentrated within specific biochemical cascades, notably “Glycerophospholipid metabolism” and “D-amino acid metabolism” (Figure 7D, left). This suggests that bone loss is accompanied by distinct lipid and gut-derived metabolic dysregulation. After BBR intervention, the differential metabolites were primarily enriched in the FoxO signaling pathway and ABC transporters (Figure 7D, right), reflecting the global restorative effect of BBR on metabolic homeostasis. We next focused on the 107 characteristic metabolic markers to identify the key pathways affected by BBR (Figure 7E). The results highlighted three major metabolic networks: (1) Remodeling of lipid and glycerophospholipid metabolism: Functional mapping showed that these differential features were heavily clustered within the metabolic networks of polyunsaturated fatty acids (PUFAs), distinctly highlighting the cascades governing linoleic, arachidonic and α-linolenic acids. These observations demonstrate that BBR effectively intervenes in the remodeling of the cell membrane glycerophospholipid pool under conditions of bone loss. (2) Regulation of microbiota–host co-metabolism: Significant enrichment of typical non-host or gut-derived pathways, such as the biosynthesis of aminoglycoside antibiotics, D-amino acid metabolism and propanoate metabolism, provided direct evidence that BBR influenced the secondary metabolism of the gut microbiota. (3) Improvement of the bone microenvironment: The enrichment of arginine and proline metabolism, as well as β-alanine metabolism, further suggested a potential role for BBR in promoting bone matrix (collagen) synthesis and alleviating oxidative stress within the microenvironment.

### 3.6. Correlation Analysis of the Gut Bacteria–Metabolite–Bone Axis

**Figure 8:**
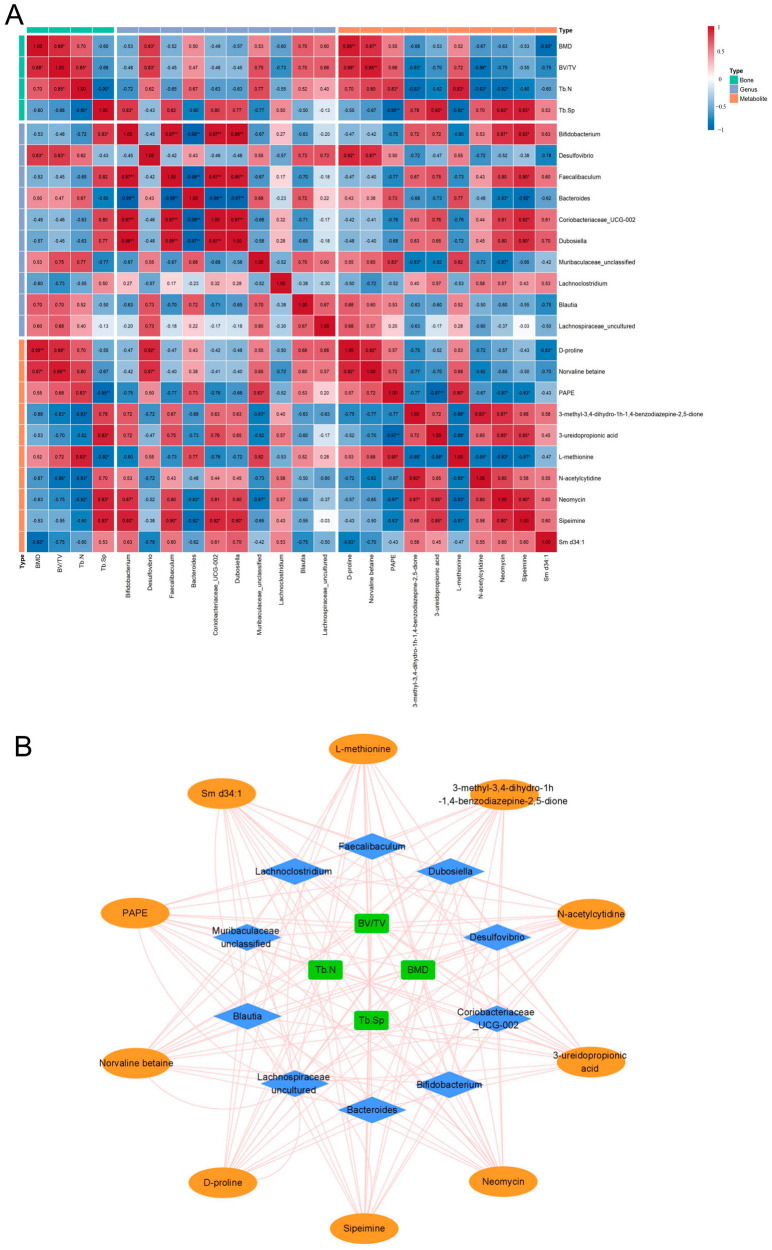
Correlation analysis. (**A**) Heatmap of the correlation between gut bacteria, metabolites, and skeletal parameters. Values represent Spearman correlation coefficients (r). Red and blue colors indicate positive and negative correlations, respectively. * *p* < 0.05, ** *p* < 0.01. (**B**) Network diagrams of gut bacteria, differential metabolites, and skeletal parameters. Orange nodes denote metabolite, blue nodes illustrate gut bacteria, and green nodes indicate skeletal parameters.

Based on the significant regulatory effects of BBR on bone phenotype, gut bacteria, and serum metabolic profiles, we further integrated four core skeletal metabolism parameters (BMD, BV/TV, Tb.N, Tb.Sp), the top 20 dominant bacterial genera in abundance, and 107 differential metabolites using Spearman correlation analysis (Figure 8), aiming to explore the potential synergistic regulatory network of the “gut–metabolite–bone” axis. The correlation analysis (Figure 8A) revealed that although *Bacteroides*, Muribaculaceae_unclassified, *Blautia*, and Lachnospiraceae_uncultured showed positive trends with BMD and BV/TV, only *Desulfovibrio* was significantly and positively correlated with these bone indicators (*p* < 0.05). Notably, lipid molecules [such as 2-arachidonoyl-1-palmitoyl-sn-glycero-3-phosphoethanolamine (PAPE)], exhibited a strong positive correlation with Tb.N (*p* < 0.05) and a significant negative correlation with Tb.Sp (*p* < 0.05). In addition to glycerophospholipids, several amino acid-related metabolites, including D-proline and Norvaline betaine, were significantly and positively correlated with BMD and BV/TV (*p* < 0.05). Furthermore, a diverse group of metabolites, including 3-methyl-3,4-dihydro-1H-1,4-benzodiazepine-2,5-dione, N-acetylcytidine, Neomycin, and Sm d34:1, showed robust negative correlations with BMD, BV/TV, and Tb.N (*p* < 0.05). Conversely, 3-ureidopropionic acid, Neomycin, and Sipeimine were found to be positively correlated with the bone resorption-related parameter Tb.Sp (*p* < 0.05). Collectively, these statistical correlations provide a multi-dimensional metabolic landscape that aligns with the improved bone phenotype observed following BBR intervention.

At the “gut bacteria–metabolite” interaction level, *Desulfovibrio* showed a highly statistically significant positive correlation with various differential metabolites, including D-proline (r = 0.92, *p* < 0.01) and Norvaline betaine (r = 0.87, *p* < 0.01). Additionally, metabolites Neomycin and alkaloid Sipeimine showed significant positive correlations with multiple bacterial genera such as *Bifidobacterium*, *Faecalibaculum*, Coriobacteriaceae UCG-002 and *Dubosiella* (*p* < 0.05). At the same time, metabolite PAPE and dominant bacterial genera such as Muribaculaceae_unclassified, *Bacteroides*, and *Desulfovibrio* also exhibited a certain positive correlation trend, but only with Muribaculaceae_unclassified reached a significant level. Moreover, the association network diagram (Figure 8B) further visually presents the complex relationship among “gut bacteria–metabolite–bone”. In summary, the BBR intervention caused synchronous fluctuations in the abundance of specific bacterial groups (such as *Desulfovibrio*, *Bacteroides*) and the levels of lipids (PAPE, Sm d34:1) and amino acid metabolites (D-proline) in the circulatory system, and these change trends are logically consistent with the improvement of bone quality.

### 3.7. Metabolite Origin Tracing and Source-Based Functional Analysis

**Figure 9:**
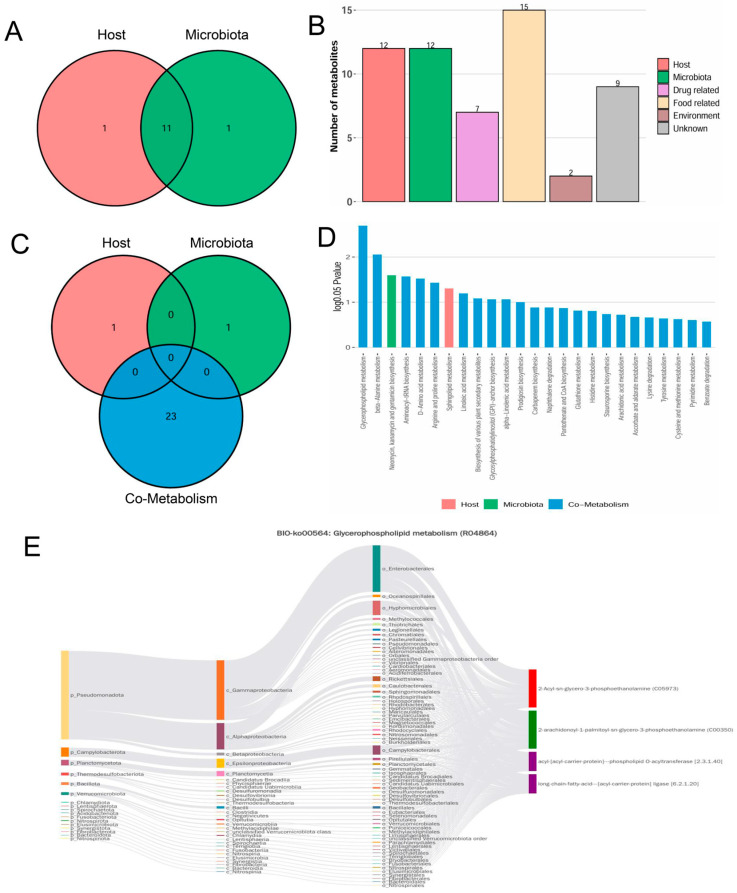
Origin analysis of the top 25 significant differential metabolites. (**A**) Intersection mapping of metabolic provenance. (**B**) Quantitative distribution classifying the sources of these biochemicals. (**C**) Venn diagram of pathway enrichment analysis. (**D**) Histogram of pathway enrichment analysis. (**E**) Bio-Sankey network diagram of Glycerophospholipid metabolism (Reaction ID: R04864).

To identify the origins and biological functions of the metabolites, we utilized the MetOrigin platform to perform source tracking analysis on the top 25 differential metabolites most strongly correlated with bone parameters. As shown in Figure 9A,B, these metabolites had diverse origins, comprising 11 bacteria-host co-metabolites, 1 bacteria-specific metabolite, and 1 host-specific metabolite. Additionally, the profile included 15 food-related, 7 drug-related, and 2 environment-related metabolites. Functional enrichment analysis revealed glycerophospholipid metabolism as the most significantly enriched pathway (Figure 9C,D). This pathway was predominantly driven by microbiota–host co-metabolites. Subsequent analysis using a microbiome–metabolite Sankey network unveiled that reaction R04864 (the PAPE synthesis reaction) served as a critical node bridging the core microbiota and the metabolic profile (Figure 9E). Within this reaction network, members of the phylum Thermodesulfobacteriota, specifically the genus *Desulfovibrio* and its related genera, which were significantly enriched by BBR as previously described, possess the enzymatic capacity for PAPE synthesis. This suggests that the increased abundance of *Desulfovibrio* after BBR treatment is functionally consistent with the enhanced biosynthetic potential of PAPE.

## 4. Discussion

The pathological basis of bone loss lies in the imbalance of bone remodeling homeostasis, driven predominantly by impaired osteogenic activity alongside accelerated osteoclastic bone degradation. Long-term glucocorticoid exposure, the leading cause of bone loss, not only directly impairs the activity of bone-metabolizing cells but also disrupts gut microecological homeostasis and the host metabolic network [4]. This disruption creates a vicious cycle wherein intestinal dysbiosis drives systemic metabolic derangements, which in turn exacerbates defective bone turnover [24]. Due to the favorable safety profile and suitability for long-term intervention dietary-derived natural functional ingredients, they have emerged as a prominent research hotspot in food science and nutrition. As a natural bioactive compound derived from medicinal and edible plants, BBR has been proven to exert diverse physiological functions, including modulating glucose and lipid homeostasis, ameliorating the gut microecology, and protecting skeletal health [15,25].

BBR has been reported to effectively alleviate OP and bone loss in vivo [19]. Insights derived from network pharmacology, coupled with molecular docking simulations, provided a preliminary computational prioritization of the potential targets through which BBR may exert its osteoprotective effects. The identified candidate targets (AKT1, TP53, and SRC) point toward the potential involvement of AKT signaling in modulating osteoblastic function [26]. It is well-established that phosphatidylinositol, a critical subtype of glycerophospholipids, serves as a key upstream molecule of the PI3K/AKT signaling axis, and its metabolic homeostasis is vital for maintaining osteoblast function [27]. More importantly, functional enrichment analyses pointed toward a molecular convergence within the PI3K complex and associated lipid metabolic circuitries. Notably, previous studies have predominantly focused on BBR’s regulation of inflammatory pathways within the local bone microenvironment [28].

The in vivo animal experiments showed that BBR intervention alleviated DEX-induced bone loss. Micro-CT and H&E staining provided morphological evidence that BBR partially preserved bone microarchitecture, while ELISA assays showed increased serum PINP and decreased CTX-1 levels. Western blotting further confirmed upregulated protein expression of COL1 and BMP2. Together, these results suggest a potential modulatory role of BBR in balancing bone formation and resorption. Notably, compared to the “antagonistic” local therapeutic strategies of traditional bisphosphonates (e.g., alendronate) that exclusively target bone tissue [29], BBR presents a systemic modulatory potential that may be linked to the remodeling of the metabolic microenvironment [30]. Furthermore, as a natural medicine-food homologous bioactive factor, BBR is generally considered to have a favorable safety profile [31,32], potentially offering a complementary approach to conventional pharmaceuticals for long-term management.

Given that the gut–bone axis modulates skeletal homeostasis through microbial metabolites and immune signaling, the anti-osteoporotic effects of BBR may be closely linked to its regulation of intestinal microecology [33]. GIOP is frequently accompanied by intestinal barrier impairment and the translocation of pathogenic bacteria, which triggers systemic low-grade inflammation and accelerates bone resorption [34,35]. In our study, BBR intervention reduced opportunistic pathogens and enriched specific genera linked to lipid metabolism, particularly *Desulfovibrio*. This implies that this taxon might represent a potential microbial marker associated with BBR-mediated gut–bone crosstalk. Serum metabolomics further identified glycerophospholipid metabolism as the key metabolic pathway underlying the skeletal benefits of BBR. Untargeted profiling revealed marked dysregulation of glycerophospholipid metabolism in the model group, a well-recognized metabolic signature of OP [36,37]. Notably, BBR alleviated DEX-induced metabolic disorders, particularly those involving polyunsaturated fatty acids and glycerophospholipid metabolism. By improving these systemic lipid metabolic abnormalities, BBR may contribute to the restoration of metabolic homeostasis, which is conducive to osteogenic differentiation [38], which aligns with our in silico prediction that the PI3K/AKT signaling axis may be a downstream target of BBR [39]. Growing evidence has demonstrated that the gut microbiota modulates bone homeostasis through downstream metabolites, including lipids and small-molecule molecules, forming a functional “microbiota–metabolite–bone” regulatory axis [40,41]. Together, these findings suggest a potential mechanism by which BBR attenuates glucocorticoid-induced bone loss by regulating the gut microbiota–glycerophospholipid metabolic axis.

Integrative multi-dimensional assessments combined with MetOrigin-based compound tracing revealed crosstalk between the gut bacteria and glycerophospholipid metabolism, supporting a regulatory network of “BBR-*Desulfovibrio*-PAPE-Bone”. Historically, *Desulfovibrio* has been considered to have potential pro-inflammatory properties [42]. However, recent studies have revealed its “Janus-faced” dual nature [43]. As a major gut sulfate-reducing bacterium, *Desulfovibrio* can produce moderate hydrogen sulfide (H_2_S); notably, previous studies have shown that appropriate H_2_S levels are contribute to alleviating OP by inducing osteoblastic differentiation and mitigating bone loss, which lays a foundation for explaining the potential osteogenic role of this genus [44,45]. Our study found that BBR treatment increased the abundance of *Desulfovibrio*, which was associated with improved bone microarchitecture and alleviation of DEX-induced bone loss. This observation, combined with the known effects of H_2_S on bone metabolism, provides a plausible mechanistic hypothesis for the association between this genus and bone mass recovery. Since H_2_S concentrations were not measured, this proposed mechanism remains inferential and requires further validation.

More importantly, *Desulfovibrio* exhibited a strong synergistic covariation with core serum glycerophospholipid metabolites (such as PAPE). Although source tracking analysis indicated that some of the identified metabolites were derived from food, drugs, or environmental sources, functional enrichment analysis still identified glycerophospholipid metabolism as the most significantly perturbed pathway. Importantly, this pathway was predominantly driven by microbiota–host co-metabolites rather than exogenous compounds, thereby supporting its relevance to endogenous crosstalk between the gut microbiota and host. The MetOrigin analysis suggests that *Desulfovibrio* may be involved in the synthesis of PAPE through the R04864 reaction. While classical *Desulfovibrio* strains do not directly annotate the corresponding acyltransferase gene, its wide conservation in related genera of the Desulfurococcaceae family suggests this function may exist [46]. Overall, BBR may overcome its low bioavailability by acting as an intestinal “fermentation substrate” [47], thereby promoting *Desulfovibrio*-mediated synthesis of the key glycerophospholipid PAPE.

In this study, BBR was administered to DEX-induced bone loss mice at 100 mg/kg daily, translating to an approximate human equivalent dose (HED) of 8.1 mg/kg [48] via body surface area normalization. While difficult to achieve through regular diet alone, this level is easily achieved with standard commercial BBR supplements (typically 500 mg per capsule), and daily 500–1500 mg BBR supplementation is clinically safe and effective for improving metabolic parameters [49,50]. Therefore, this study provides an experimental basis for using BBR as a routine nutritional supplement for maintaining bone health, and also lays the foundation for the targeted development of functional foods in the future. Consistent with existing literature reporting the beneficial effect of BBR on bone loss [38], our findings further support this potential, while an appropriate increase in sample size (such as the number of samples for micro-CT detection) would help to further clarify the role of BBR. We have revealed a potential mechanism of BBR in bone protection that is partially derived from predictive multi-omics analyses, and further experimental validation (e.g., fecal microbiota transplantation) could be performed to verify the causal link underlying the observed associations. Expanding our investigations to additional bone loss models, such as postmenopausal models, would not only improve the generalizability of our conclusions but also favor the translational development of BBR as a promising bone-protective intervention.

## 5. Conclusions

**Figure 10:**
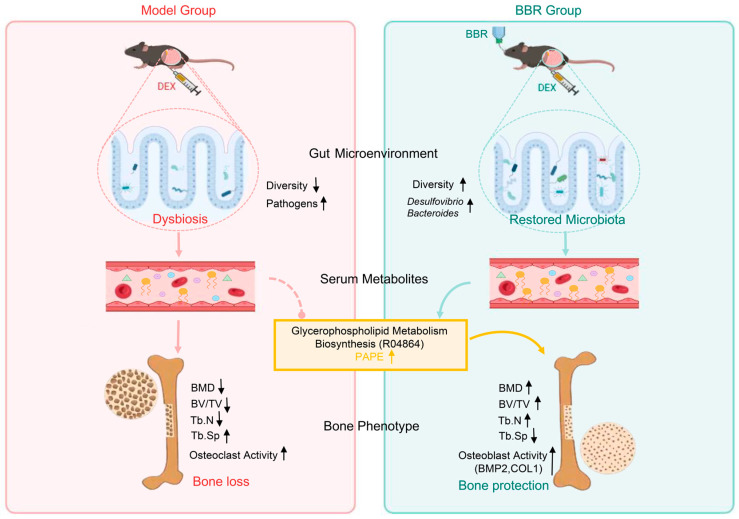
Schematic diagram illustrating the potential protective mechanism of BBR against DEX-induced bone loss via regulation of the gut microbiota–glycerophospholipid metabolic axis. (**Left**) Panel (Red): Depicts the pathological state induced by DEX, characterized by gut dysbiosis and severe bone loss. (**Right**) Panel (Green): Demonstrates that BBR intervention may remodel the gut microbiota, enhance glycerophospholipid biosynthesis, and promote osteoblast differentiation, which may contribute to increased bone mass and attenuated bone loss.

In summary, by integrating in vivo efficacy evaluation with multi-omics analysis, this study demonstrates that the natural alkaloid BBR effectively increases BMD and ameliorates DEX-induced bone loss. Our findings suggest that BBR may achieve these effects by restoring gut microecological homeostasis, notably through enriching the genus *Desulfovibrio*. Such microbiota remodeling is associated with elevated biosynthesis of the key metabolite PAPE, which may contribute to the recovery of disturbed glycerophospholipid metabolism in the host (Figure 10). These results provide a theoretical basis for further investigation of BBR, supporting its potential as a promising bioactive ingredient for the development of dietary interventions and bone-protective functional foods.

## Data Availability

The raw data supporting the conclusions of this article, including 16S rRNA sequencing reads and metabolomics raw profiles, are openly available in Figshare at https://doi.org/10.6084/m9.figshare.31866280. Further inquiries regarding other study data can be directed to the corresponding authors.

## Supplementary Materials

The following supporting information can be downloaded at https://www.mdpi.com/article/10.3390/foods15081325/s1, Figure S1. Western blot analyses of three independent biological replicates corresponding to Figure 3B.

## Abbreviations

Abbreviation	Definition
AKT1	Protein kinase B1
ANOVA	Analysis of variance
ASVs	Amplicon sequence variants
BBR	Berberine
BMD	Bone mineral density
BMP2	Bone morphogenetic protein 2
BV/TV	Bone volume fraction
CC	Cellular component
COL1	Collagen type I
CTX-1	C-terminal telopeptide of type I collagen
DEX	Dexamethasone
ELISA	Enzyme-linked immunosorbent assay
GO	Gene Ontology
H&E	Hematoxylin and eosin
KEGG	Kyoto Encyclopedia of Genes and Genomes
Micro-CT	Micro-computed tomography
NMDS	Non-metric multidimensional scaling
OP	Osteoporosis
OPLS-DA	Orthogonal partial least squares discriminant analysis
P1NP	N-terminal propeptide of type I procollagen
PAPE	2-arachidonoyl-1-palmitoyl-sn-glycero-3-phosphoethanolamine
PCA	Principal component analysis
PCoA	Principal coordinate analysis
PI3K	Phosphatidylinositol 3-kinase
PPI	Protein–protein interaction
PUFA	Polyunsaturated fatty acid
QC	Quality control
ROI	Region of interest
SD	Standard deviation
SRC	SRC proto-oncogene
Tb.N	Trabecular number
Tb.Sp	Trabecular separation
TP53	Tumor protein p53
