# Supplementary material for: Berberine Attenuates Glucocorticoid-Induced Bone Loss in Mice: Associated with the Gut Microbiota–Glycerophospholipid Metabolic Axis
Source: Foods. 2026 Apr 10;15(8):1325. doi: 10.3390/foods15081325 (PMC13115423; doi:10.3390/foods15081325)

Berberine Attenuates Glucocorticoid-Induced Bone Loss in Mice: Associated with the Gut Microbiota–Glycerophospholipid Metabolic Axis

Suzhen Chao [1,2,3,†], Shengyuan Li [1,3,†], Jimin Zhong [1,3], Xinyi Peng [1], Yang Li [1,3], Min Shi [4], Xing Hu [5,6,*] and Bo Liu [1,3,*]

Part 1: Independent biological replicates

Repeat 1 **Repeat 2** **Repeat 3**

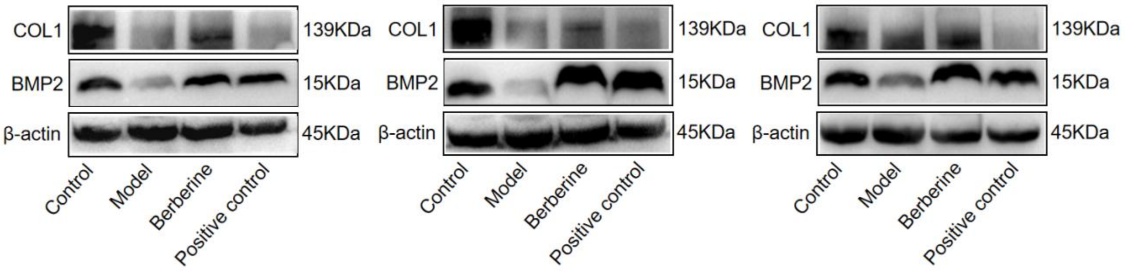

Supplementary Figure 1. Western blot analyses of three independent biological replicates corresponding to Figure 3B.

Part 2: Original Scans

Target: COL1 (139 KD)

Repeat 1：

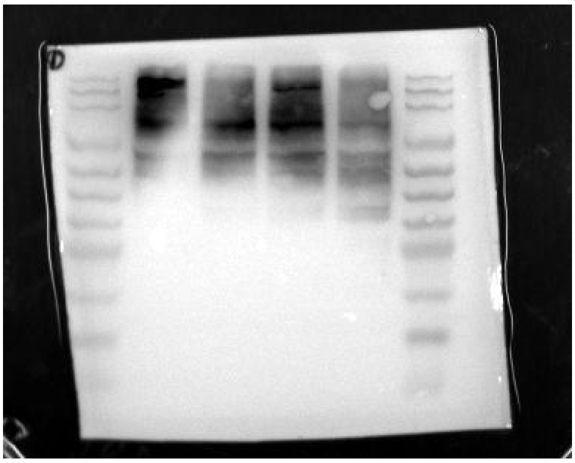

Repeat 2：

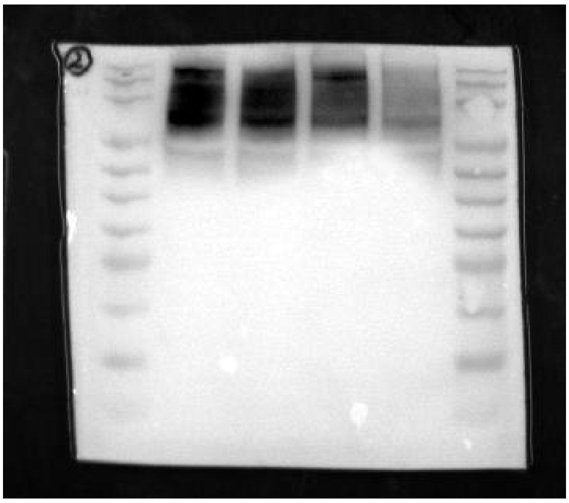

Repeat 3：

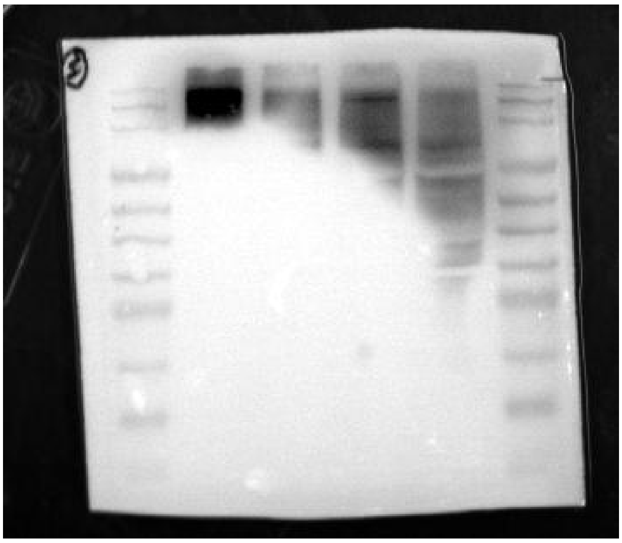

Target: BMP2 (15 KD)

Repeat 1：

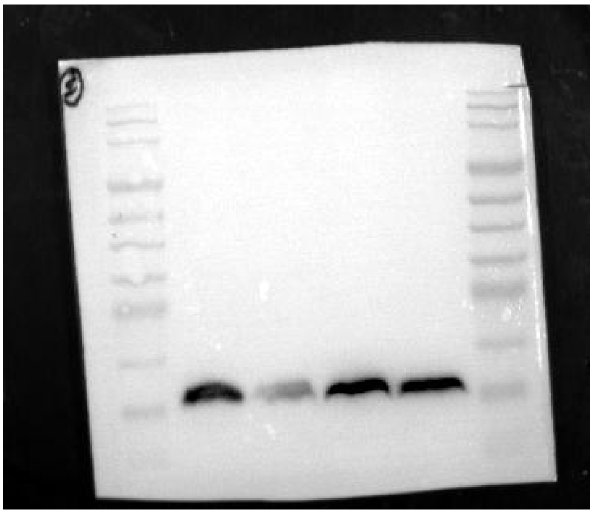

Repeat 2：

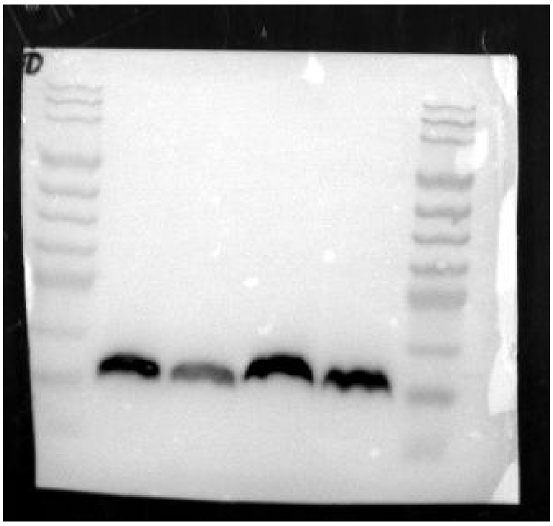

Repeat 3：

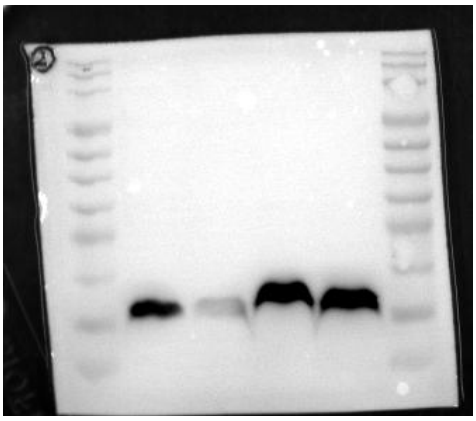

Loading Control: β-actin (45 KD)

Repeat 1：

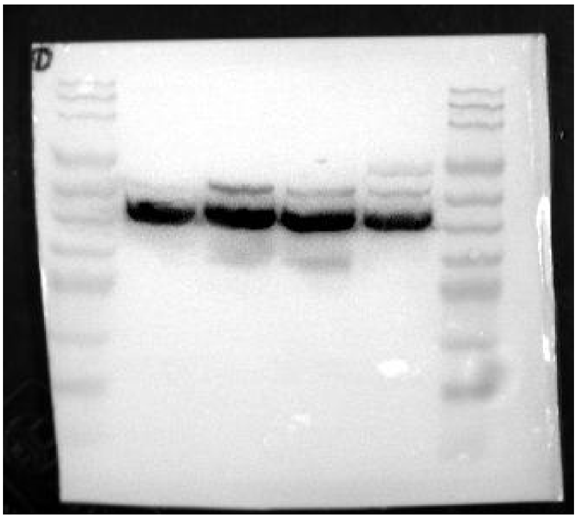

Repeat 2：

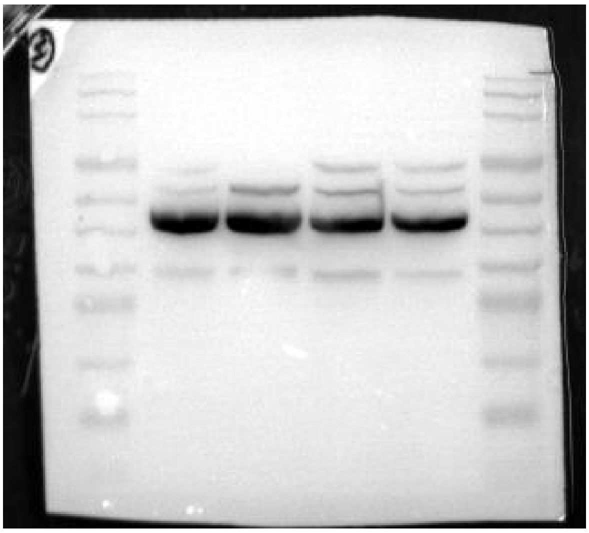

Repeat 3：

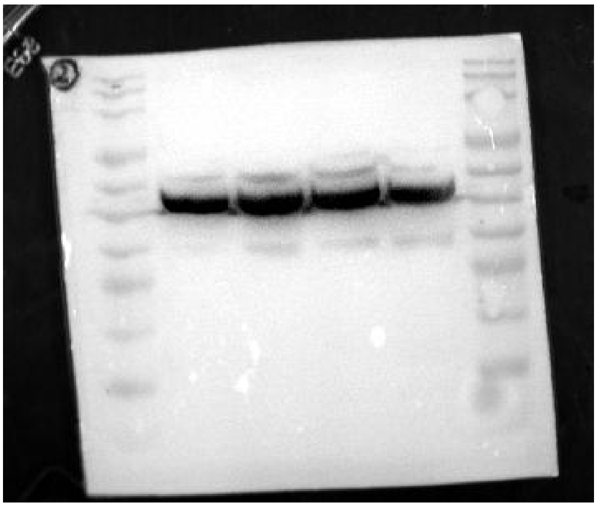

Western blot marker image

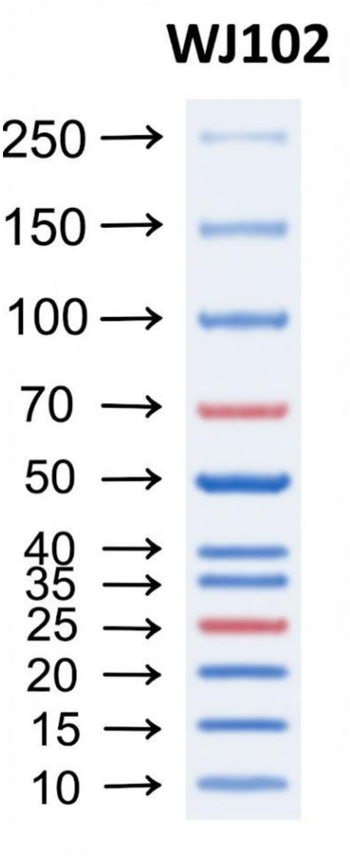

Supplement: Supplementary file 1 [file foods-15-01325-s001.zip › foods-4212729-supplementary.pdf]
